# Reimbursement pricing for new medical devices in Japan: Is the evaluation of innovation appropriate?

**DOI:** 10.1002/hpm.2719

**Published:** 2018-12-14

**Authors:** Makoto Tamura, Shohei Nakano, Takuma Sugahara

**Affiliations:** ^1^ Medical Technology Policy Research Institute American Medical Devices and Diagnostics Manufacturers' Association Tokyo Japan; ^2^ Medical Device Strategy Institute Japan Association for the Advancement of Medical Equipment Tokyo Japan; ^3^ Graduate School of Health Sciences International University of Health and Welfare Tokyo Japan; ^4^ Department of Economics Hosei University Tokyo Japan

**Keywords:** reimbursement pricing, Japan, medical devices, foreign average price, evaluation of innovation

## Abstract

**Objectives:**

In Japan, strong reimbursement pricing control measures for existing medical device products have rendered new medical device reimbursement pricing critical for manufacturers. Few studies have been conducted on this aspect; therefore, this paper (1) clarifies whether evaluation of innovation is appropriate or not and (2), if not, investigates its background.

**Methods:**

In this research, 319 C1/C2 government decisions for new medical devices in the 10 years from April 2008 to March 2018 were analyzed. Evaluation of innovation was considered in terms of the reimbursement price, as well as the foreign average price ratio.

**Results:**

Considering the degree of evaluation of innovation, the average premium rate for the similar function category comparison method was 10.2% during 2008 to 2010 (this means the newly set reimbursement price was 10.2% higher than that of corresponded exiting categories); it declined consistently thereafter, to 3.2% during 2016 to 2018. Moreover, evaluation of innovation by the foreign average price (FAP) ratio was 1.04 in 2008 to 2010, consistently decreasing to 0.88 in 2016 to 2018. The period from product approval to the non‐Special Designated Treatment Material (non‐STM) (a part of technical fee) price listing is much longer than that of the STM (own reimbursement price) listing.

**Conclusion:**

Several reasons were considered for the decline in innovation evaluation: (1) the lowering of the FAP upper limit ratio, (2) the possibility that there was not enough evidence at the time of price listing, (3) and the more rigorous standards to create a new separate functional category. However, some aspects were attributable to reimbursement system reform.

## 1 INTRODUCTION

Japan's medical device market of approximately 2.9 trillion yen, as of 2016, constitutes about 6.3% of the National Health Insurance's (NHI) total medical expense. This ratio did not change significantly from 1984 to 2010, and the correlation between total medical expenses and the medical device market was significantly high.^1^ Hence, it can be said that the medical device market is fully controlled with respect to medical expenses.

One of the reasons for the financial control of the device market is the existence of several strong reimbursement price control measurements for existing products; these include a price reduction mechanism based on the average selling price to hospitals/clinics and a price reduction rule to set a ceiling relative to the foreign average price (FAP).

When manufacturers want to retain profits to some extent for R&D investment, new products need to be launched with a high price, as the reimbursement price of existing products always decrease. In the past, price revisions (reductions) were made every other year; however, the government has recently tried to revise prices annually (not yet decided for medical devices).

Hence, reimbursement pricing for new medical devices is critical for manufacturers, and the medical device industry has advocated the importance of the pricing rule for new medical devices, in other words the evaluation of innovation (as expressed in terms of the reimbursement price decided by the government).

Despite the current importance of new device reimbursement pricing as described above, it has been the subject of few empirical studies. Hence, this paper has the following three objectives:

1. Assess whether the evaluation of innovation is appropriate or not.
2. If not, investigate its background.
3. Compare the reimbursement process and issues between several product categories, for example Special Designated Treatment Material (STM) and non‐STM categories, as described later.

This paper first describes the determination of reimbursement pricing for new medical devices (Section 2) and outlines the method of the study (Section 3). Section 4 presents the results, which are discussed in Section 5. Section 6 concludes.

## 2 OVERVIEW OF JAPAN'S MEDICAL DEVICE REIMBURSEMENT POLICY

In Japan's NHI system, which realized universal coverage in 1961, medical fees are determined on a fee‐for‐service basis. The Diagnosis Procedure Combination system was introduced in inpatient medical care, with a fixed payment per day in many acute care hospitals; however, surgery reimbursement is based on a fee for service out of a fixed fee. Therefore, many medical implant devices used during surgery are still paid on a fee‐for‐service basis.

Two major types of reimbursement rules exist for medical devices. The first rule determines the prices for individual medical devices, for example, implant and disposal device types such as pacemakers and artificial joints (hereafter, STM). The second rule incorporates the price as part of the technical fee for diagnostic devices such as computerized tomography (CT) or magnetic resonance imaging (MRI) scanners, or medical devices to be used repeatedly (hereafter, non‐STM).

STM medical devices have certain set reimbursement prices. However, prices are set for each functional category instead of per brand name; these categories are similar in structure, purpose of use, and clinical efficacy.^2^ For example, stents for coronary arteries have four categories: general, emergency treatment, restenosis constrain (ie, drug‐eluting stent or DES), and a bioabsorbable/restenosis constrain (ie, bioresorbable vascular scaffold (BVS)).

Non‐STM devices are reimbursed as part of the technical fee. In the case of an imaging diagnostic device, for instance, a fixed amount is paid to the medical institution for MRI use. Payment is therefore not associated with the equipment itself, but a medical fee is paid for the act of diagnostic imaging.

**Figure 1:**
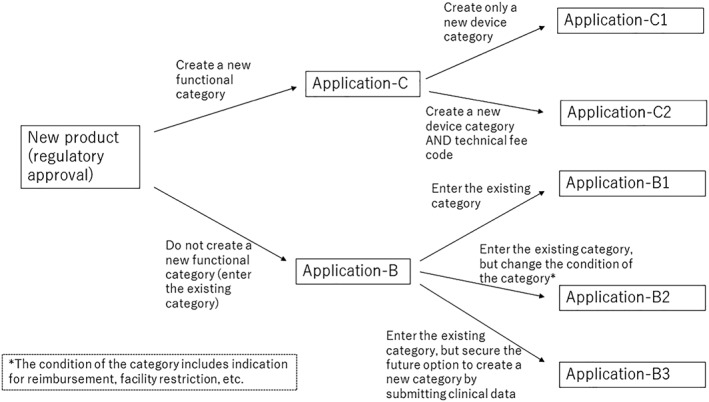
Process for reimbursement listing application (STM)

**Figure 2:**
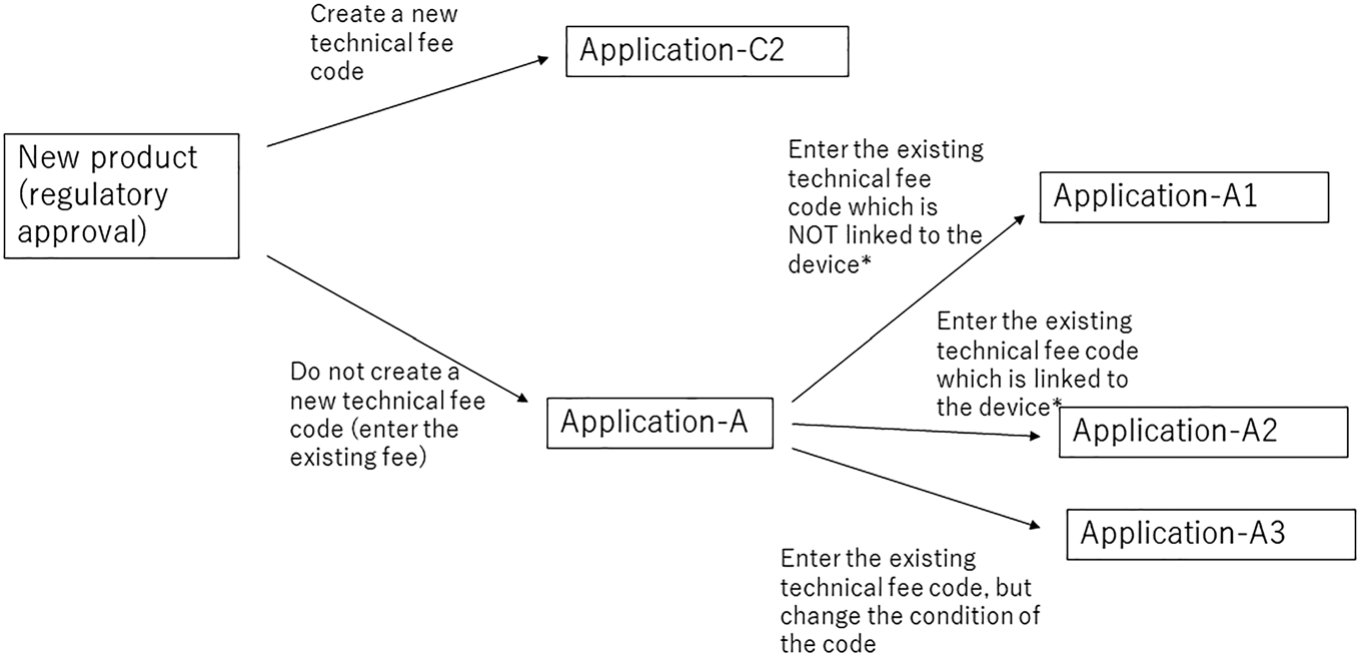
Process for reimbursement listing application (non‐STM)

When a company acquires regulatory approval for a new product, it submits a reimbursement request dossier (in Japanese: HokenTekiyo Kibosho) to the Ministry of Health, Labour and Welfare (MHLW).^2^ When manufacturers want to create a new device category or technical fee, they need to submit a C1 or C2 application (more detail is provided in Figures 1 and 2).

C1 and C2 applications differ as follows: C1 is a method to request the establishment of a new functional category of STM devices, but not a technical fee (for example, surgical fee), when using the STM device. C2 is an application to request a new technical fee, and comprises either of two options^1^: cases where a new STM category is requested at the same time^2^ and cases where only a technical fee is requested.

**Figure 3:**
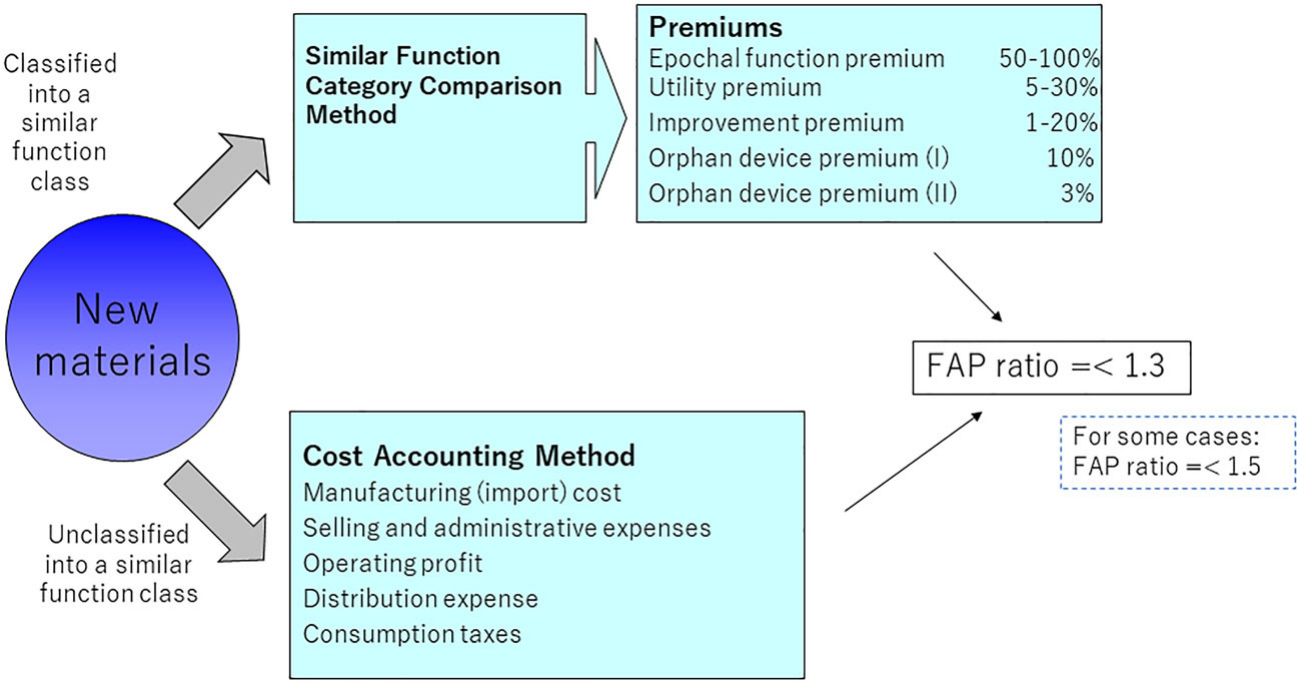
Rule for creating a new category (STM) title page [Colour figure can be viewed at wileyonlinelibrary.com]

The price setting rules for new STM products are determined in detail. If a similar function category exists, the similar function category comparison method is used; if not, the cost accounting method is used (Figure 3).^2^

In the case of the similar function category comparison method, several kinds of premiums exist: the epochal function premium (in Japanese: Kakkisei‐kasan), utility premium (Yuyosei‐kasan), improvement premium (Kairyo‐kasan), and orphan premium (Shijosei‐kasan; Figure 3). To achieve the epochal function premium, a new product has to meet with three conditions: a clinically effective new product structure, higher effectiveness/safety compared with an existing category, and improvement of the treatment method. For the utility premium, any of the three conditions should be met. For the improvement premium, an incremental improvement aspect should exist, such as lower invasiveness for patients. The epochal function premium provides a rate that is within 50% to 100% of the premium price rate, the utility premium is within 5% to 30%, and the incremental premium is within 1% to 20%. The premium price rate indicates the additional price (%) that is added to the reimbursement price of existing categories; for example, when the existing category price is $100 and the new category price is $120, the premium price rate is 20%.

For the cost accounting method, the reimbursement price is calculated as the manufacturing cost (import cost) plus selling and administrative expenses, as well as other relevant expenses; it is based on the predicted sales quantity.

In both methods, the upper limit is 1.3 times the FAP (includes the prices for the United States, United Kingdom, Germany, France, and Australia; the upper limit can be up to 1.5 times the FAP, if certain requirements are met). This means that when the FAP is $100, the reimbursement price cannot be set higher than $130 (or $150, with certain conditions).

Non‐STM devices are not subject to such detailed rules. It is first decided whether a new technical fee should be set up; thereafter, until the time of revision of the whole medical fee once every 2 years, the technical fee of similar technology is temporarily adopted. A new technical fee shall be set at revision.

For both STM and non‐STM devices, the process up to the insurance listing is clearly defined. It includes the submission of a dossier, a hearing by MHLW, a hearing by the expert panel (Hoken Iryo Zairyo Tou Senmon Soshiki), a decision on the final proposal, an appeal complaint against the proposed decision (if necessary), and approval by the Chuikyo (the government reimbursement advisory council).

This medical device reimbursement system gradually evolved since the mid‐1990s; its present framework was created in 2002. Furthermore, at the biannual medical fee revision, reform such as strengthening the system for premium prices or the reduced upper limit of the FAP ratio (the Japanese price divided by the FAP) was executed.

## 3 METHOD

Companies must submit application C1/C2 to set up a reimbursement price for a medical device that is not evaluated by the conventional functional category or technical fee. Chuikyo's records on the MHLW website indicate that it approved 364 devices as C1/C2 from April 2008 to March 2018. Excluding dental products, this study analyzed 319 cases of newly approved medical devices classified as C1/C2 to clarify the current situation and identify issues. Dental products were excluded, since there is a significant difference in the revision rules for reimbursement prices; the medical supply system and the market size of dental products comprise less than 10% of all medical devices.^3^

Information on C1/C2 is available from the MHLW website.^4^ Product regulatory approval dates were obtained from JAAME SEARCH (a paid database).^5^

The information used for analysis included the determined classification of the NHI price listing (C1/C2); the regulatory approval date; the reimbursement listing date; the method of determining the reimbursement price (for STM devices, either the similar function category comparison method or the cost accounting method, and for non‐STM devices, the technical fee); the type and rate of the premium price (in the case of the similar function category comparison method); and the FAP ratio.

## 4 RESULTS

### 4.1 Overview

From 2008 to 2018, 319 cases were listed as C1/C2; C1 and C2 comprised 184 and 135 cases, respectively (see Table 1). All C1 cases involved STM devices. Of the C2 cases, technical fees were set with STM's medical device price setting for 74 cases, and only the technical fee was set for 61 cases (non‐STM devices).

**Table 1:** Overview of reimbursement decisions by period

	Total Number of Cases		Determined Category				Calculation Method							Premium Price Method (Similar Function Category Comparison Method)							Number of Approvals (Reference)^a^
							STM					Non‐STM									
Period			C1		C2		Similar Function Category Comparison Method		Cost Accounting Method		Others (Change of Existing Function Category)	Technical Fee		Epochal Function Premium	Utility Premium		Improvement Premium		No Addition		
2008‐2010	29	(100.0)	24	(82.8)	5	(17.2)	18	(62.1)	10	(34.5)	0	1	(3.4)	0	7	(38.9)	6	(33.3)	5	(27.8)	91
2010‐2012	74	(100.0)	37	(50.0)	37	(50.0)	42	(56.8)	12	(16.2)	3	17	(23.0)	0	20	(47.6)	8	(19.0)	14	(33.3)	104
2012‐2014	105	(100.0)	65	(61.9)	40	(38.1)	53	(50.5)	27	(25.7)	6	19	(18.1)	0	13	(24.5)	32	(60.4)	8	(15.1)	174
2014‐2016	67	(100.0)	39	(58.2)	28	(41.8)	26	(38.8)	30	(44.8)	0	11	(16.4)	0	6	(23.1)	14	(53.8)	6	(23.1)	114
2016‐2018	44	(100.0)	19	(43.2)	25	(56.8)	17	(38.6)	13	(29.5)	1	13	(29.5)	0	3	(17.6)	7	(41.2)	7	(41.2)	74
Total	319	(100.0)	184	(57.7)	135	(42.3)	156	(48.9)	92	(28.8)	10	61	(19.1)	0	49	(31.4)	67	(42.9)	40	(25.6)	557

a The number of “new medical devices” and “improved medical devices (with clinical data)” in the approval review category of PMDA (“improved medical devices without clinical data” and “me‐too devices” are excluded).

The number of C1/C2 cases over this decade varied significantly, depending on the 2‐year bracket; most cases (105) were during 2010 to 2012, whereas the least cases (29) were during 2008 to 2010. C1 comprised 82.8% of the total number of cases in 2008 to 2010; it decreased to 43.2% during 2016 to 2018, whereas C2 comprised 56.8% of cases. The number of C2 cases constantly increased.

The same trend was found for the number of approvals of “new medical devices” and “improved devices (in case clinical data are reviewed)”—which constitute regulatory review categories for the same period as the main source of C1 and C2. Approvals peaked during 2010 to 2012; the number of cases in other years were low, and the correlation coefficient between the number of these approvals and C1/C2 cases was as high as 0.89 (since there is a time lag between approval and reimbursement listing, these two items do not correspond exactly).

### 4.2 Reimbursement calculation method for STM devices

The similar function category comparison method for STM devices was used for 156 cases (ie, about 50% of cases); the cost accounting method for STM devices was used for 92 cases, and the non‐STM method was used for 61 cases.

The annual trend (see Table 1) indicates that, while the similar function category comparison method accounted for more than 50% of cases from 2008 to 2012, it remarkably decreased after 2014. At the same time, the cost accounting method and the non‐STM method increased. An analysis of the number of cases by premium out of the similar function category comparison method indicates that the utility premium comprised more than half during 2008 to 2012 and decreased afterward, whereas the improvement premium accounted for more than half from 2012 to 2016. In 2016 to 2018, the total number of cases decreased, but the number of “no premium price” relatively increased; it was only 15% in 2012 to 2014, but exceeded 40% in 2016 to 2018.

While “no premium price” has no meaning in terms of the immediate reimbursement price, it represents to some extent a meaningful decision for companies, because it enables the new provision of a single functional category at the subsequent price revision; this new provision is not affected by the actual selling price of existing products or by FAP (the reimbursement prices of existing categories are revised every other year, based on the actual selling price or FAP).

There was no case of epochal function premium in the observed 10 years. Since 2002, when the framework of the current system was formulated, one case of epochal function premium was approved, namely the DES in 2004.

### 4.3 Evaluation of innovation

The average premium rate for STM devices' similar function category comparison method was 5.8% over the 10 years (see Table 2). This means that the newly set reimbursement price was on average 5.8% higher than that of corresponded exiting categories. The reimbursement price was 10.4% in 2008 to 2010, but declined consistently to 3.2% in 2016 to 2018.

**Table 2:** Evaluation of innovation by 2‐year bracket

Period	Premium Rate (Similar Function Category Comparison Method)	FAP Ratio		
		Total	Similar Function Category Comparison Method	Cost Accounting Method
2008‐2010	10.4%	1.04	0.96	1.15
2010‐2012	6.5%	1.00	0.95	1.02
2012‐2014	5.1%	0.96	0.94	0.95
2014‐2016	4.8%	0.89	0.86	0.92
2016‐2018	3.2%	0.88	0.81	0.96
Total	5.9%	0.95	0.92	0.97

Moreover, the newly set reimbursement price of STM devices was 0.95 times the FAP; in other words, it was, on average, 95% of the FAP. This value decreased gradually since 2008 to 2010 to 1.04 times in 2008 to 2010 and 0.88 times in 2016 to 2018; the rate of the premium showed a similar trend.

Considering the FAP ratio by calculation method, the cost accounting calculation method was higher: 0.97 times for the cost accounting method and 0.92 times for the similar function category comparison method.

### 4.4 Period from regulatory approval to reimbursement listing

The average period from regulatory approval to reimbursement listing was 279.4 days overall, of which C1 constituted 234.4 days and C2 constituted 340.8 days (see Table 3). It varied depending on the method of calculation: 203.1 days for the similar function category comparison method and 258.7 days for the cost accounting method. For technical fee, the average period was 431.8 days.

**Table 3:** Average duration from the date of approval to reimbursement listing

			Average Duration from the Date of Approval to Reimbursement Listing
All			279.4
Decision category		C1	234.4
		C2	340.8
Calculation method	STM	Similar function category comparison method	203.1
		Cost accounting method	258.7
		Others (change of existing function category, etc)	731.6
	Non‐STM	Technical fee	431.8
Period		2008‐2010	200.2
		2010‐2012	261.2
		2012‐2014	316.2
		2014‐2016	188.3
		2016‐2018	413.4

The tendency increased over time when the 2‐year bracket is considered; however, it was shorter in 2014 to 2016; it can therefore not be considered a consistent trend.

The standard administrative processing period defined by the MHLW is 4 months from the first day of the month following submission of the dossier for C1 and 5 months for C2. According to Nakano's survey,^6^ since the average period from approval to submission of the reimbursement dossier is about 1 month, the average period from the current regulatory approval to insurance reimbursement seems to be within the standard administrative processing period.

## 5 DISCUSSION

The issues and related policy reform are discussed from two perspectives: “appropriate evaluation for innovation” and “transparency/predictability of the system.”

### 5.1 Appropriate evaluation of innovation

Although it is not easy to judge whether the evaluation of innovation is appropriate, two perspectives are considered.

#### 5.1.1 Change in evaluation over time and its background

The rate of the premium price and the FAP ratio are both decreasing over time. It is almost certain that the evaluation of new products submitted in C1/C2 has been declining.

This can be interpreted in several ways. First, Japan's financial situation is severe, with slow economic growth and an aging population; the pressure to control social security expenditure, especially medical cost, is very high. These factors have a direct and indirect influence, and the FAP ceiling ratio of the C1/C2 insurance reimbursement gradually decreased between 2000 and the 2010s. The initial upper limit of 2.0 times declined to 1.3 times. As the upper limit has been lowered, it seems that the average FAP ratio also decreased. However, when the upper limit was reduced to 1.3 times in 2016, the upper limit was kept as 1.5 times in the case of the epochal function premium and the utility premium of 10% or more, which shows consideration for innovation evaluation.

Second, it is possible that the MHLW and Chuikyo's policy on the premium price and evaluation is changing. The expert panel's July 2017 proposal indicated that there is no need to newly set the functional category for “replaceable products.”^7^ The expert panel implied that, if the new product was merely replaced with old products, it would mean that new and old products would have the same patient targets, the same usage, and so on; therefore, new a category does not have to be set. In an associated case at the April 2018 revision, the replaced new product category was merged with the old category (there was another case by a similar policy in the April 2016 revision). For example, the pacemaker and implantable cardioverter defibrillator (ICD) categories, for use in MRI scans, used to have a premium price; following revision, they were merged with the pacemaker and ICD categories, which cannot be used in MRI.^2^

Conventionally, new functional categories and premium prices were set up in response to a slight functional improvement as a result of product improvement, such as when invasiveness was lowered or the procedure was simpler and/or safer for the health care professional, even if clinical data were insufficient. This no longer seems to be accepted.

A new system was established in April 2018, namely, application B3 in Table 1.^7^ In this application, even if clinical data are not enough, the premium price can be approved subject to a time limit, without creating a new functional category. Therefore, it is possible that the evaluation of improved products, which will replace existing products, will improve in future.

Third, it is possible that the data necessary for reimbursement price evaluation are becoming insufficient due to the policy change of the medical regulatory authorities in the late 2000s. The authorities provided early approval to reduce “device lag” (the much slower introduction of products into Japan, compared with other countries), even without adequate clinical efficacy data.

The lack of evidence regarding reimbursement of medical devices at the time of the reimbursement listing has been widely pointed out in Europe and the United States, and has been addressed by methods such as Coverage with Evidence Development.^8^, ^9^ A similar system, called “Challenge Application,” was introduced in April 2018 to address industry requests.^7^ This system creates a new functional category, or obtains a new premium price, by submitting new clinical data after the reimbursement listing. If this system works effectively, even if there is a tendency for data to become insufficient at the reimbursement listing, an appropriate evaluation may be done in the future. It is necessary to continue to closely monitor the situation.

#### 5.1.2 Sufficiency of the reimbursement price

This section considers whether or not the newly allocated reimbursement price, as an absolute value, is sufficient.

Nakano considers it as insufficient that the average rate of the premium price of 54 items evaluated by the similar function category comparison method in 2008 to 2012 was 7%,^10^ since the reimbursement price of the existing products is annually reduced by 6% to 7% on average, based on MHLW's selling price survey. This is done even if 7% of the premium price is added, if it is assumed that development started 4 years ago, and if the premium price needs to be increased by 13% to 14% to add up to the original price. The development period can be longer. According to this study's results, the average premium rate further declines to below 7%, and it is most recently 3.2%. From Nakano's perspective, companies' economic incentives for new product development are lacking.

This survey indicates that, compared with foreign average prices, the cost accounting and similar function category comparison methods are 0.97 times and 0.92 times the FAP, respectively. Considering that it is required to be equal to or more than the FAP, the cost accounting method is about appropriate; however, the similar function category comparison method can be considered as below standard. Assessing this by reimbursement year, prices determined by the similar function category comparison method have been consistently decreasing from 2008 to 2018, but not those by the cost accounting method (Table 2). The latter is generally considered to be at an appropriate level in comparison with FAP.

As discussed earlier, innovation evaluation may be insufficient due to a lack of evidence at the time of reimbursement listing. In the case of the cost accounting method, if efficacy and safety are confirmed by regulatory approval, the evidence of comparison with existing products is not strictly required (since there is no existing functional category to be compared, the cost accounting method is chosen). It is therefore possible that evaluation of the average price up to the FAP could be obtained. At the same time, the higher FAP ratio of the cost accounting method can be explained as the cost of product development and launch, including training for health care professionals, product registry, and others.

A health technology assessment (HTA) decision will be required on whether the price is appropriate in the future (in Japan, HTA is called “cost effectiveness evaluation”). The Chuikyo committee was established in Japan in 2012, and the HTA trial evaluation was initiated in 2016 for some products.

Even if Japan's reimbursement price for a certain product was higher than foreign prices, it might be cost‐effective for the Japanese medical environment, and vice versa. As is often pointed out, the HTA of a medical device has many difficulties due to insufficient clinical evidence, which is based on methods such as randomized control trials, the influence of the learning curve, frequent product improvement, and others. If such difficulties are to be properly solved, it will be important to introduce the HTA concept appropriately.^11^

### 5.2 Transparency and predictability of STM and non‐STM products

For STM and non‐STM products, the transparency and predictability of pricing differ substantially. There are various detailed rules on how price is determined for each of the similar function category comparison and cost accounting methods of STM products (however, many parts still require subjective judgment by the decision maker).

Only the cost of the device and its medical economic usefulness need to be submitted for non‐STM products; no rule exists on how to set the technical fee based on submitted materials.

These difficulties associated with non‐STM products occur in the period from product approval to price listing. In the case of STM devices, the similar function category comparison method averaged 203.1 days and the cost accounting method averaged 258.7 days, while the average of non‐STM (technical fee) cases was as long as 431.8 days. In some cases, companies may not even know how to request a price listing, and it seems that there are instances in which the MHLW found it difficult to determine.

While STM cases allow for official participation by companies in pricing rule determination and actual reimbursement request processes, non‐STM cases relate to a technical fee; here, Chuikyo's involvement revolves around payer and the medical professional perspective to decide a technical fee; company involvement is inevitably restricted.

However, taking into consideration that companies are obliged to stably supply products once they have been insured, the process for both STM and non‐STM devices would be more balanced if the company was involved to some extent.

## 6 CONCLUSION

This study empirically analyzed Japan's current process of new medical device reimbursement (C1/C2) over the past 10 years. Results indicate that innovation evaluation gradually declined. This tendency was specifically observed for the similar function category comparison method, compared with the cost accounting method. There are three potential main reasons for this^1^: the fact that the FAP upper limit ratio has been lowered,^2^ the possibility that there was not enough evidence at the time of price listing,^2^ and the more rigorous standards used in creating a new separate functional category. During the 2016 to 2018 reform, three new rules—the two‐stage system of the FAP upper limit ratio, a Challenge Application, and a B3 application—were implemented to respond to these problems.

Further, this paper has shown that the reimbursement listing process of non‐STM devices is not clear compared with that of STM devices and that the period from product approval to price listing of non‐STM devices is much longer than that of STM devices.

This research has some limitations. First, data were based on results determined by C1/C2 case evaluations. Even when a company applies for C1/C2, there are cases that are not accepted and, as a result, new products are reimbursed in the existing STM or technical fee categories. Inclusion of these cases in the research sample could indicate that innovation assessment has been less appropriately done. We may survey individual companies in the future to show the results for entire reimbursement request situations.

Second, innovation evaluation gradually declined. This study did not verify the possibility that products with high efficacy and safety are no longer being developed.

Third, FAP represents the price that manufacturers report to the government; it is not the actual reimbursed price. However, the Japanese government uses this value for price reductions, and believes it to be reliable. Since we interpreted FAP as a trend rather than an absolute value, the analysis with respect to FAP should be acceptable.

The Japanese reimbursement system for new devices seems to maintain a high level of transparency and predictability; however, the pricing method is not clear for non‐STM products (as compared with STM products), and it takes relatively long to make reimbursement decisions. This aspect can represent a challenge in future.
